# Analytical Prediction for Nonlinear Buckling of Elastically Supported FG-GPLRC Arches under a Central Point Load

**DOI:** 10.3390/ma14082026

**Published:** 2021-04-17

**Authors:** Zhicheng Yang, Airong Liu, Jie Yang, Siu-Kai Lai, Jiangen Lv, Jiyang Fu

**Affiliations:** 1 College of Urban and Rural Construction, Zhongkai University of Agriculture and Engineering, Guangzhou 510225, China; zhicheng.yang@zhku.edu.cn (Z.Y.); lvjiangen77@163.com (J.L.); 2 Wind and Vibration Engineering Research Center, Guangzhou University, Guangzhou 510006, China; liuar@gzhu.edu.cn; 3 School of Engineering, RMIT University, P.O. Box 71, Bundoora, VIC 3083, Australia; jie.yang@rmit.edu.au; 4 Department of Civil and Environmental Engineering, The Hong Kong Polytechnic University, Kowloon, Hong Kong, China; sk.lai@polyu.edu.hk; 5 Hong Kong Branch of National Rail Transit Electrification and Automation Engineering Technology Research Center, The Hong Kong Polytechnic University, Kowloon, Hong Kong, China

**Keywords:** limit point buckling, bifurcation buckling, elastically supported FG-GPLRC arch, analytical solutions

## Abstract

In this paper, we present an analytical prediction for nonlinear buckling of elastically supported functionally graded graphene platelet reinforced composite (FG-GPLRC) arches with asymmetrically distributed graphene platelets (GPLs). The effective material properties of the FG-GPLRC arch are formulated by the modified Halpin–Tsai micromechanical model. By using the principle of virtual work, analytical solutions are derived for the limit point buckling and bifurcation buckling of the FG-GPLRC arch subjected to a central point load (CPL). Subsequently, the buckling mode switching phenomenon of the FG-GPLRC arch is presented and discussed. We found that the buckling modes of the FG-GPLRC arch are governed by the GPL distribution pattern, rotational restraint stiffness, and arch geometry. In addition, the number of limit points in the nonlinear equilibrium path of the FG-GPLRC arch under a CPL can be determined according to the bounds of successive inflexion points. The effects of GPL distribution patterns, weight fractions, and geometric configurations on the nonlinear buckling behavior of elastically supported FG-GPLRC arches are also comprehensively discussed.

## 1. Introduction

Functionally graded material (FGM) structures, characterized by a continuous change in the material compositions along one or multiple directions, have attracted extensive attention from both research and industrial communities owing to their excellent stiffness and strength-to-weight properties as compared with homogeneous composite structures [1,2,3]. To better understand the performance of FGM structures in practical engineering applications, researchers have conducted a series of investigations on the structural behavior of FGM structures. For instance, Ke et al. [4] presented analytical solutions for the nonlinear vibration responses of FGM beams with different end supports and discussed the influence of bending-stretching coupling on the nonlinear vibration of FGM beams. Librescu et al. [5] analyzed the vibration and stability of FGM thin-walled beams under a high-temperature environment. Yan et al. reported analytical solutions for the dynamic instability [6] and dynamic responses [7] of FGM beams with open-edge cracks. They investigated the crack depth and location effects on the mechanical behavior of such beams. Nguyen et al. [8] studied the mechanical buckling of stiffened FGM plates by using the finite element method. They found that the addition of stiffeners to the FGM plate could significantly reduce the weight of the FGM plate. Chen et al. [9] investigated the imperfection sensitivity in the nonlinear vibration of initially stressed FGM plates. According to their results, the effects of the initial stress, geometric imperfection, and volume fraction index were quite significant on the nonlinear vibration behavior of the FGM plate. Hao et al. [10] employed an asymptotic perturbation method to analyze the nonlinear oscillations, bifurcations, and chaotic motions of FGM plates. More relevant studies to investigate the significant performance of FGM structures could be found from the open literature [11,12,13,14,15,16,17].

Graphene, as an emerging high-performance nanofillers, has attracted considerable attention in aerospace, mechanical, thermal, and electrical engineering fields. It has been demonstrated by researchers that the reinforced performance of graphene nanoplatelets (GPLs) is significantly superior to other reinforcement materials. Adding a low concentration of GPLs can improve the stiffness and strength of reinforced composites significantly [18,19,20,21,22,23]. By introducing GPLs to FGM materials, novel FG GPLs-reinforced composite (FG-GPLRC) structures have been developed recently and have since attracted extensive attention in both research and engineering communities [24]. Yang and his co-workers conducted pioneering studies on the mechanical behaviors of FG-GPLRC structures, such as beams [25,26,27,28], shells [29,30], and plates [31,32]. Focusing on the stability analysis of FG-GPLRC arches, the authors devoted extensive efforts to the investigation of such structures, including the characteristics of nonlinear static buckling, dynamic buckling, and free vibration for FG-GPLRC arches with different boundary conditions and external loads [33,34,35,36,37,38,39]. In addition, Liu et al. [40] analyzed the nonlinear behavior and stability of functionally graded porous (FGP) arches reinforced by GPLs and obtained the critical buckling load under a uniform load. Zhao et al. [41] discussed the linear buckling, fundamental frequency, and dynamic instability of porous arches using an analytical method. Li et al. [42] further investigated the mechanics of the confined porous arches by including the thermal effect. Hitherto, to the best of our knowledge, far too little attention has been paid to the nonlinear buckling behavior of elastically supported FG-GPLRC arches with asymmetric distributed GPLs under a central point load (CPL).

Therefore, to fill this research gap, the nonlinear buckling behavior of elastically supported FG-GPLRC arches under a CPL is investigated in this study. The effective material properties of the FG-GPLRC arch are formulated by the modified Halpin–Tsai micromechanical model, because the Young’s modulus of the FG-GPLRC arch predicted by this model agreed well with the experimental results [19]. Subsequently, the principle of virtual work is employed to derive the nonlinear buckling load for the limit point buckling and bifurcation buckling modes, from which the critical geometric parameters to identify the buckling load and the number of limit points can also be determined. In addition, the influences of GPL distribution patterns, GPL weight fractions, geometric configurations, as well as the flexibility of the rotational constraints on the nonlinear buckling behavior of the arch are discussed in detail. The bifurcation stability criteria presented in this work can provide essential information for the structural/material design of FG-GPLRC arches that have great potential in various engineering applications, for example, arch-shaped micro-electromechanical devices as sensors and transducers [43], and dielectric elastomer actuators as lightweight speakers [44,45]. In addition, the presence of analytical solutions is useful to engineers and researchers for benchmarking the convergence and validity of numerical methods for arch buckling analysis.

## 2. Effective Material Properties

**Figure 1:**
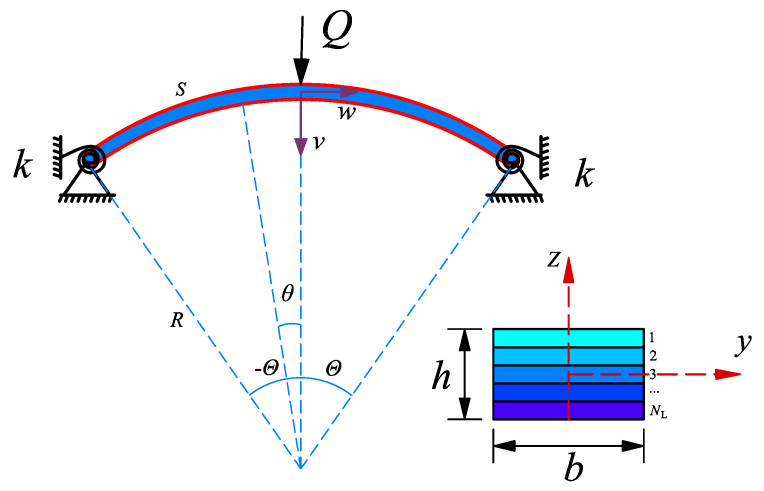
Configuration and coordinate system of an elastically supported functionally graded graphene platelet reinforced composite (FG-GPLRC) arch.

In Figure 1, we consider an elastically restrained FG-GPLRC arch with multiple layers, *N*_L_, under a CPL *Q*. The rectangular cross section of the arch is *b* × *h* (width × thickness).

It is assumed that the reinforcement of GPLs is uniformly distributed in the isotropic polymer matrix, so each individual GPLRC layer can be regarded as an isotropic and homogeneous material. The variation of GPLs is continuous along the arch thickness in accordance with the power law distribution. The GPL volume fraction of the *k*th GPLRC layer $V_{\mathrm{GPL}}^{k}$ is formulated as follows:

(1) $$V_{\mathrm{GPL}}^{k}=V_{\mathrm{GPL}}^{*}\left[\frac{1}{2}+{\left(\frac{k-1}{N_{L}-1}\right)}^{n}\right]$$

where *N*_L_ is the total number of the GPLRC layer, and *n* is the power law index that characterizes the distribution of GPLs. When *n* = 0, it corresponds to a uniform distribution of GPLs reinforcements in the thickness direction. $V_{\mathrm{GPL}}^{*}$ is the total GPL volume fraction which can be determined using:

(2) $$V_{\mathrm{GPL}}^{*}=\frac{W_{\mathrm{GPL}}}{W_{\mathrm{GPL}}+\left(\rho_{\mathrm{GPL}}/\rho_{m}\right)\left(1-W_{\mathrm{GPL}}\right)}$$

where *W*_GPL_ is the GPLs weight fraction and $\rho_{\mathrm{GPL}}$ and $\rho_{m}$ are the mass densities of GPLs and matrix, respectively.

According to the modified Halpin–Tsai micromechanical model [43], the effective Young’s modulus *E^k^* of the *k*th GPLRC layer is given by

(3) $$E^{k}=\frac{3\left(1+\xi_{L}\eta_{L}V_{\mathrm{GPL}}^{k}\right)}{8\left(1-\eta_{L}V_{\mathrm{GPL}}^{k}\right)}\times E_{m}+\frac{5\left(1+\xi_{T}\eta_{T}V_{\mathrm{GPL}}^{k}\right)}{8\left(1-\eta_{T}V_{\mathrm{GPL}}^{k}\right)}\times E_{m}$$

with

(4) $$\eta_{L}=\frac{\left(E_{\mathrm{GPL}}/E_{m}\right)-1}{\left(E_{\mathrm{GPL}}/E_{m}\right)+\xi_{L}},\;\eta_{T}=\frac{\left(E_{\mathrm{GPL}}/E_{m}\right)-1}{\left(E_{\mathrm{GPL}}/E_{m}\right)+\xi_{T}}\;\xi_{L}=2\left(a_{\mathrm{GPL}}/b_{\mathrm{GPL}}\right)\times \xi_{T}/2,\;\xi_{T}=2\left(b_{\mathrm{GPL}}/t_{\mathrm{GPL}}\right)$$

where *E*_GPL_ and *E*_m_ are the Young’s moduli of GPLs and matrix, respectively. *a*_GPL_, *b*_GPL_, and *t*_GPL_ are the length, width, and thickness of GPLs, respectively.

The Poisson’s ratio $\upsilon^{k}$ of each GPLRC layer is determined by the rule of mixture as follows:

(5) $$\upsilon^{k}=\upsilon_{\mathrm{GPL}}V_{\mathrm{GPL}}^{k}+\upsilon_{m}\left(1-V_{\mathrm{GPL}}^{k}\right)$$

where $\upsilon_{\mathrm{GPL}}$ and $\upsilon_{m}$ are the Poisson’s ratios of GPLs and matrix, respectively.

**Figure 2:**
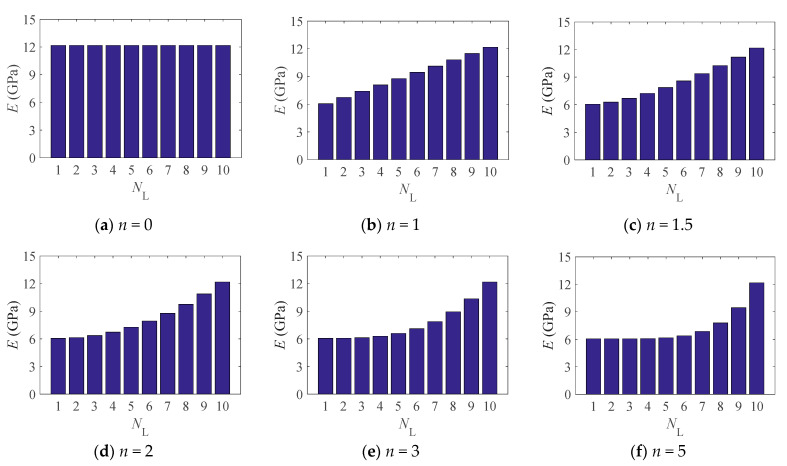
Effective Young’s modulus of an elastically restrained FG-GPLRC arch.

Consider GPLs with the material properties *E*_GPL_ = 1010 GPa, $\rho_{\mathrm{GPL}}=1062.5\mathrm{kg}/m^{3}$, $\upsilon_{\mathrm{GPL}}=0.186$, *a*_GPL_ = 2.5 µm, *b*_GPL_ = 1.5 µm, and *t*_GPL_ = 1.5 nm, and epoxy (polymer matrix) with *E*_m_ = 3 GPa, $\upsilon_{m}=0.34$, and $\rho_{m}=1200\mathrm{kg}/m^{3}$ [25], the effective Young’s moduli of the FG-GPLRC arch with 10 GPLRC layers under various GPLs distributions (different power law indices) are shown in Figure 2.

## 3. Mathematical Modeling

As shown in Figure 1, an elastically supported FG-GPLRC arch with an angle 2Θ, a radius *R,* and an arc length *S* under the effect of a CPL is studied in this section. The stiffness of the elastic rotational constraints at both ends is *k*, the radial and axial displacement of the arch are *v* and *w*, respectively, and *θ* is the angular coordinate. By using the Donnell’s shallow shell theory [39], the nonlinear strain-displacement relations for the FG-GPLRC arch are adopted as follows:

(6) $$\varepsilon ={\tilde{w}}^{\prime }-\tilde{v}+\frac{{{\tilde{v}}^{\prime }}^{2}}{2}-\frac{z}{R}{\tilde{v}}^{\prime \prime }\;\mathrm{with}\;\tilde{v}=v/R\;\mathrm{and}\;\tilde{w}=w/R$$

where ${\left(\;\right)}^{\prime }=d(\;)/d\theta$.

According to the principle of virtual work, the governing equation is established as:

(7) $$\delta W=\int_{-\Theta }^{\Theta }Rb\int_{-h/2}^{h/2}E^{k}\varepsilon \delta \varepsilon \;dzd\theta -\int_{-\Theta }^{\Theta }\delta_{D}\left(\theta \right)QR\delta \tilde{v}\;d\theta +\sum_{i=\pm \Theta }k{\tilde{v}}_{i}^{\prime }\delta {\tilde{v}}_{i}^{\prime }=0$$

where $\delta_{D}\left(\theta \right)$ is the Dirac delta function [27]. 

Substituting Equation (6) into Equation (7), one obtains

(8) $$\delta W=\int_{-\Theta }^{\Theta }\left[-NR\left(\delta {\tilde{w}}^{\prime }-\delta \tilde{v}+{\tilde{v}}^{\prime }\delta {\tilde{v}}^{\prime }\right)-M\delta {\tilde{v}}^{\prime \prime }-\delta_{D}\left(\theta \right)QR\delta \tilde{v}\right]\;d\theta +\sum_{i=\pm \Theta }k{\tilde{v}}_{i}^{\prime }\delta {\tilde{v}}_{i}^{\prime }=0$$

with the axial force

(9) $$N=-A_{11}\left({\tilde{w}}^{\prime }-\tilde{v}+\frac{{{\tilde{v}}^{\prime }}^{2}}{2}\right)+\frac{B_{11}}{R}{\tilde{v}}^{\prime \prime }$$

and the bending moment

(10) $$M=B_{11}\left({\tilde{w}}^{\prime }-\tilde{v}+\frac{{{\tilde{v}}^{\prime }}^{2}}{2}\right)-\frac{D_{11}}{R}{\tilde{v}}^{\prime \prime }=-\left(D_{11}-\frac{B_{11}^{2}}{A_{11}}\right)\frac{{\tilde{v}}^{\prime \prime }}{R}-\frac{B_{11}}{A_{11}}N$$

where

(11) $$\left\{A_{11}\;B_{11}\;D_{11}\right\}=b\int_{-h/2}^{h/2}E^{k}\left\{1\;z\;z^{2}\right\}\;\mathrm{dz}=b\sum_{k=1}^{N_{L}}\int_{z_{k}}^{z_{k+1}}E^{k}\left\{1\;z\;z^{2}\right\}\;\mathrm{dz}.$$

As the present model is derived in accordance with the classical Euler–Bernoulli theory, so that $A_{11}$, $B_{11}$ and $D_{11}$ are *E^k^*-based constants.

From Equation (8), the boundary conditions for the elastically supported FG-GPLRC arch are given by

(12) $$\tilde{v}\left(\Theta \right)=\tilde{v}\left(-\Theta \right)=\tilde{w}\left(\Theta \right)=\tilde{w}\left(-\Theta \right)=0\;-M\left(\Theta \right)+k{\tilde{v}}_{\Theta }^{\prime }=-M\left(-\Theta \right)-k{\tilde{v}}_{-\Theta }^{\prime }=0$$

Substituting Equations (9) and (10) into Equation (8), obtains the equilibrium equations as follows:

(13) $${\left(NR\right)}^{\prime }=0$$

(14) $$NR+{\left(NR{\tilde{v}}^{\prime }\right)}^{\prime }-M^{\prime \prime }-\delta_{D}\left(\theta \right)QR=0$$

Inserting Equations (9), (10) and (13) into Equation (14) yields the radial equilibrium equation as follows:

(15) $$\frac{{\tilde{v}}^{iv}}{\mu^{2}}+{\tilde{v}}^{\prime \prime }=\frac{\delta_{D}\left(\theta \right)QR^{2}}{\kappa \mu^{2}}-1\;\mathrm{with}\;\kappa =D_{11}-\frac{B_{11}^{2}}{A_{11}}\;\mathrm{and}\;\mu^{2}=\frac{NR^{2}}{\kappa }$$

where κ is effective bending stiffness of the FG-GPLRC arch.

Solving Equation (11) and considering the boundary conditions of the elastically support of the arch, the analytical solution of the dimensionless radial displacement can be determined as follows:

(16) $$\begin{array}{ll} \tilde{v}= & \frac{\beta^{2}-\mu^{2}\theta^{2}}{2\mu^{2}}+\frac{\beta \left(\cos\mu \theta -\cos\beta \right)K_{1}}{\mu^{2}}+\frac{B\left(\cos\mu \theta -\cos\beta \right)K_{12}}{R}\\ & +\frac{P}{\mu^{2}\beta }\left[K_{2}\cos\mu \theta -\beta +K_{3}+H\left(\theta \right)\left(\mu \theta -\sin\mu \theta \right)\right] \end{array}$$

where β=μΘ, $B=B_{11}/A_{11}$, and α is the flexibility of the rotational constraints defined as:

(17) $$\alpha =\frac{\kappa }{kS}$$

The dimensionless CPL is defined as:

(18) $$P=\frac{QR^{2}\Theta }{2\kappa }$$

and the parameters *K*_1_, *K*_2_, *K*_3_, and *K*_12_ are given as:

(19) $$K_{1}=-\frac{2\alpha +1}{2\alpha \beta \cos\beta +\sin\beta },\;K_{2}=\frac{2\alpha \beta \sin\beta -\cos\beta +1}{2\alpha \beta \cos\beta +\sin\beta }\;K_{3}=\frac{1-\cos\beta }{2\alpha \beta \cos\beta +\sin\beta },\;K_{12}=\frac{2\alpha \beta }{2\alpha \beta \cos\beta +\sin\beta }$$

In Equation (16), H(θ) is a step function defined by [33] as:

(20) $$H\left(\theta \right)=\left\{ \begin{array}{l} -1\;\theta <0\\ 1\;\theta \geq 0 \end{array}\right.$$

Substituting Equation (16) into Equation (9), obtains the nonlinear equilibrium relationship as:

(21) $$A_{1}P^{2}+B_{1}P+C_{1}=0$$

where *A*_1_, *B*_1_, and *C*_1_ are determined as:

(22) $$A_{1}=\Xi_{1}K_{2}^{2}-\Xi_{2}K_{2}+\Xi_{3}$$

(23) $$\begin{array}{ll} B_{1}= & 2\beta^{2}\Xi_{1}K_{1}K_{2}-\beta^{2}\Xi_{2}K_{1}-\frac{K_{2}\cos\beta }{\beta^{3}}+\frac{\sin\beta -K_{3}}{\beta^{3}}\\ & +\frac{B_{0}}{\lambda }\left[\left(2K_{2}\Xi_{1}-\Xi_{2}\right)\beta^{3}K_{12}+\frac{K_{2}\sin\beta +\cos\beta -1}{\beta^{2}}\right] \end{array}$$

(24) $$\begin{array}{ll} C_{1}= & \beta^{4}\Xi_{1}K_{1}^{2}-\frac{1}{6}+\frac{\beta^{2}r_{11}^{2}}{\lambda^{2}}+\frac{B_{0}^{2}}{\lambda^{2}}\left(\beta^{6}\Xi_{1}K_{12}^{2}+\beta K_{12}\sin\beta \right)\\ & +\frac{B_{0}}{\lambda }\left(2\beta^{5}\Xi_{1}K_{12}K_{1}+K_{1}\sin\beta +1\right) \end{array}$$

with *B*_0_ = *B*_11_/*A*_11_*h*, $r_{11}=r_{x}/h$, $r_{x}=\sqrt{\kappa /A_{11}}$, and λ is the arch geometrical parameter as

(25) $$\lambda =\frac{R\Theta^{2}}{h}=\frac{S}{h}\frac{\Theta }{2}$$

The parameters $\Xi_{1}$, $\Xi_{2}$, and $\Xi_{3}$ are given as

(26) $$\Xi_{1}=\frac{\beta -\sin\beta \cos\beta }{4\beta^{5}},\;\Xi_{2}=\frac{{\left(\cos\beta -1\right)}^{2}}{2\beta^{5}},\;\;\Xi_{3}=\frac{\sin\beta \cos\beta +3\beta -4\sin\beta }{4\beta^{5}}$$

## 4. Nonlinear Buckling Analysis

### 4.1. Limit Point Buckling

When the FG-GPLRC arch buckles in a limit point instability mode, the limit point loads may be either local maxima or local minima on the nonlinear equilibrium path, which are derived from Equation (21) as:

(27) $$A_{2}P^{2}+B_{2}P+C_{2}=0$$

with

(28) $$\left\{\begin{array}{ccc} A_{2} & B_{2} & C_{2} \end{array}\right\}=\frac{\partial }{\partial \beta }\left\{\begin{array}{ccc} A_{1} & B_{1} & C_{1} \end{array}\right\}$$

Accordingly, the solutions of the limit point load and nonlinear equilibrium path of FG-GPLRC arches under a CPL can be determined by solving Equations (21) and (27).

### 4.2. Bifurcation Buckling

When the FG-GPLRC arch buckles in a bifurcation mode, the equilibrium equation for the arch can be derived by substituting the critical states $\left\{\tilde{v}+{\tilde{v}}_{b},\;\tilde{w}+{\tilde{w}}_{b},\;N+N_{b},\;M+M_{b}\right\}$ for the bifurcation buckling into Equation (14), we obtain:

(29) $$\frac{{\tilde{v}}_{b}^{iv}}{\mu^{2}}+{\tilde{v}}_{b}^{\prime \prime }=0$$

The general solution of Equation (29) is solved as:

(30) $${\tilde{v}}_{b}=E_{1}\sin\mu \theta +E_{2}\cos\mu \theta +E_{3}\theta +E_{4}$$

where *E*_1_, *E*_2_, *E*_3_, and *E*_4_ are unknown coefficients.

Substituting the boundary conditions of the arch into Equation (30) yields the following characteristic equation for the coefficients *E*_1_, *E*_2_, *E*_3_, and *E*_4_ as:

(31) $$\left[\frac{\sin\beta }{2\alpha }+\beta^{2}\left(\sin\beta -\frac{\cos\beta }{2\alpha \beta }\right)\right]\left(\cos\beta +\frac{\sin\beta }{2\alpha \beta }\right)=0$$

From Equation (31), for the first case, it is true that the first term becomes zero, from which the critical axial force parameter $\beta_{b}$ for the bifurcation buckling is solved as:

(32) $$\beta_{b}=\eta_{b}\pi$$

and the corresponding axial force is obtained as:

(33) $$N_{b}=\frac{\mu_{b}^{2}\kappa }{R^{2}}=\frac{{\left(\eta_{b}\pi \right)}^{2}\kappa }{{\left(S/2\right)}^{2}}=N_{E2}$$

Substituting Equation (32) into Equation (21), the bifurcation buckling equation is determined as:

(34) $$A_{3}P^{2}+B_{3}P+C_{3}=0$$

where

(35) $$A_{3}=A_{1,\;\beta =\eta_{b}\pi },\;B_{3}=B_{1,\;\beta =\eta_{b}\pi },\;C_{3}=C_{1,\;\beta =\eta_{b}\pi }$$

from which the bifurcation load is solved as:

(36) $$P=\frac{-B_{3}\pm \sqrt{B_{3}^{2}-4A_{3}C_{3}}}{2A_{3}}$$

The existence of real solutions in Equation (36) requires $B_{3}^{2}-4A_{3}C_{3}\geq 0$, this condition gives a critical geometric parameter $\lambda_{b1}$ that can trigger the bifurcation buckling of the arch.

Consider the second case of Equation (31) (i.e., the second term is zero), we may obtain the critical axial force parameter $\beta_{sn}$ as follows:

(37) $$\beta_{sn}=\eta_{sn}\pi \;(sn=s1,\;s2,\;s3,\;\ldots ,)$$

Following similar procedures, a critical geometric parameter $\lambda_{sn}$ that governs the number of inflection points in the nonlinear equilibrium path of the FG-GPLRC arch can also be determined. It is worth noting that the CPL corresponding to the first (as well as the lowest) axial force parameter $\beta_{s1}$ is also the lowest buckling load of the arch whose geometric parameter is $\lambda_{s1}$. When the FG-GPLRC arch has $\lambda \leq \lambda_{s1}$, the arch becomes a slightly curved beam and does not perform typical nonlinear buckling behavior.

## 5. Numerical Studies and Discussion

**Figure 3:**
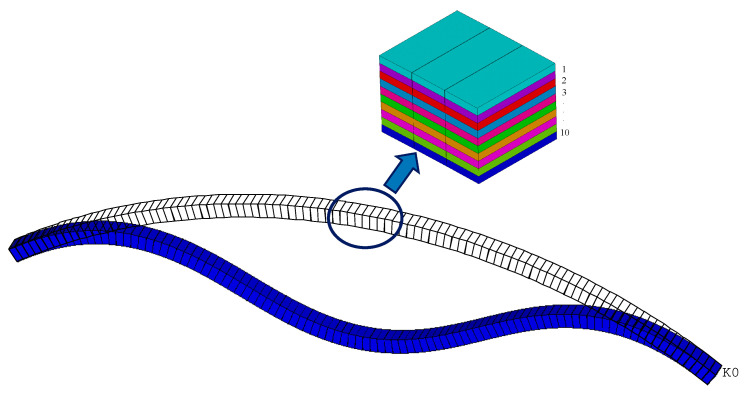
Mesh and deformation of an elastically restrained FG-GPLRC arch.

In this section, the nonlinear buckling behavior of the FG-GPLRC arch with a cross section of *b* × *h* = 0.03 m × 0.025 m and 10 GPLRC layers is investigated. The material properties of GPLs and matrix are adopted as the same in Section 2. To verify the present solutions, a finite element (FE) analysis conducted by ANSYS 17.0 is used to obtain the nonlinear equilibrium path of the FG-GPLRC arch. In numerical modeling, the SHELL181 element is used to establish a multi-layer FG-GPLRC arch model. The COMBIN14 element is adopted to model elastic end restraints whose rotational restraint stiffness *k* is defined by Equation (17). The section commands are used to define the layered structures which can provide the input options for specifying the thickness, material, and orientation of each layer. The FG-GPLRC arch model is meshed by 100 elements along the arch length direction, and 10 layers are considered to be in the thickness direction, as depicted in Figure 3.

As a large deflection and rotation may occur during the whole deformation of the arch, the NLGEOM command is used for nonlinear analysis when considering the effect of geometric nonlinearities. In addition, the arc-length method activated by the ARCLEN command is, then, used to trace the nonlinear equilibrium path of the FG-GPLRC arch with a load step of 200, as specified by the NSUBST command. By setting the minimum and maximum multipliers for the arc-length radius, the subsequent displacement and load proportional factors and the increment size can be adjusted and computed automatically using the arc-length radius.

**Figure 4:**
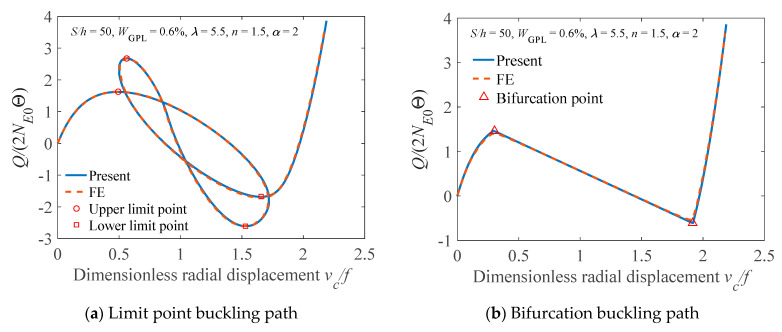
Comparison of FE results and analytical solutions.

For comparison, the FE and present results for the limit point buckling path and bifurcation buckling path are plotted in Figure 4, in which *N*_E0_ = the critical bifurcation axial force *N*_E2_ of the pure epoxy arch, and *v*_c_/*f* = the dimensionless displacement of the arch crown with *f* being the rise of the arch. It should be mention that, for triggering the bifurcation buckling behavior of the FG-GPLRC arch, an antisymmetric geometric imperfection of 0.1% arch length is introduced to the arch in the bifurcation buckling analysis. It is observed that the present analytical solutions agree very well with the FE results.

**Figure 5:**
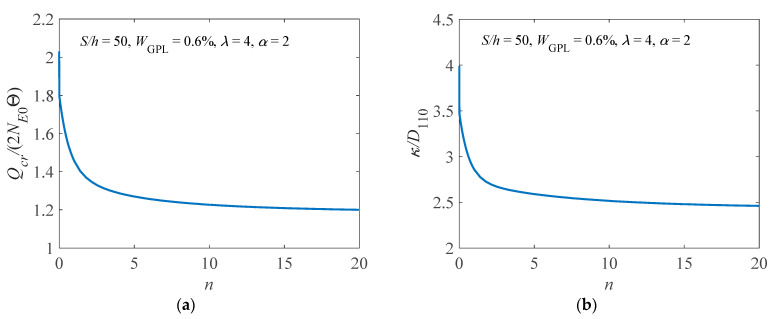
Effect of the power law index on (**a**) the buckling load and (**b**) the effective bending stiffness.

Next, we discuss the buckling load of the elastically supported arch, i.e., the limit point buckling load (the first upper limit point load) for brevity, unless stated otherwise. Figure 5 shows the influence of the power law index on the buckling load and the effective bending stiffness of the arch for α=2. In Figure 5b, “*D*_110_” is the bending stiffness of the pure epoxy arch. It is found that the buckling load and the effective bending stiffness significantly decrease as the power law index increases, but the effect of the power law index tends to be less pronounced when the power law index is higher than three. This is because a higher content of GPLs would be concentrated on the bottom and less on other layers when the FG-GPLRC arch has a higher power law index, thereby leading to a lower bending stiffness of the arch.

**Figure 6:**
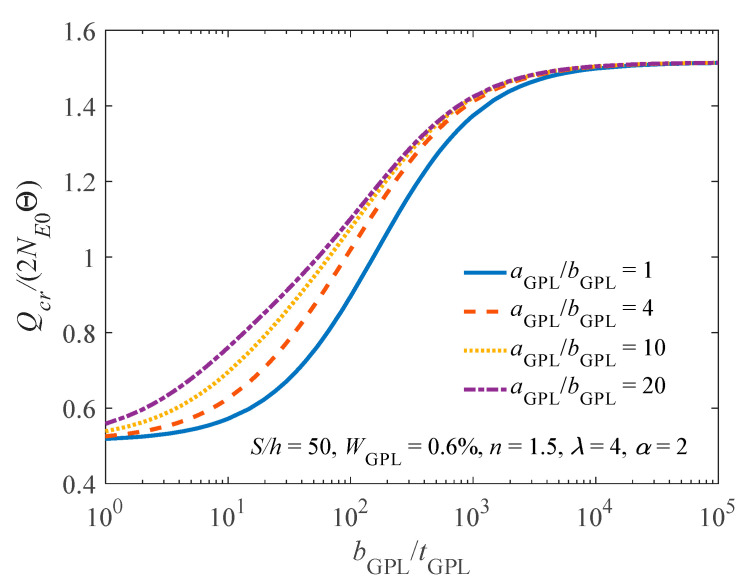
Effect of geometry and size of GPLs on the buckling load.

**Figure 7:**
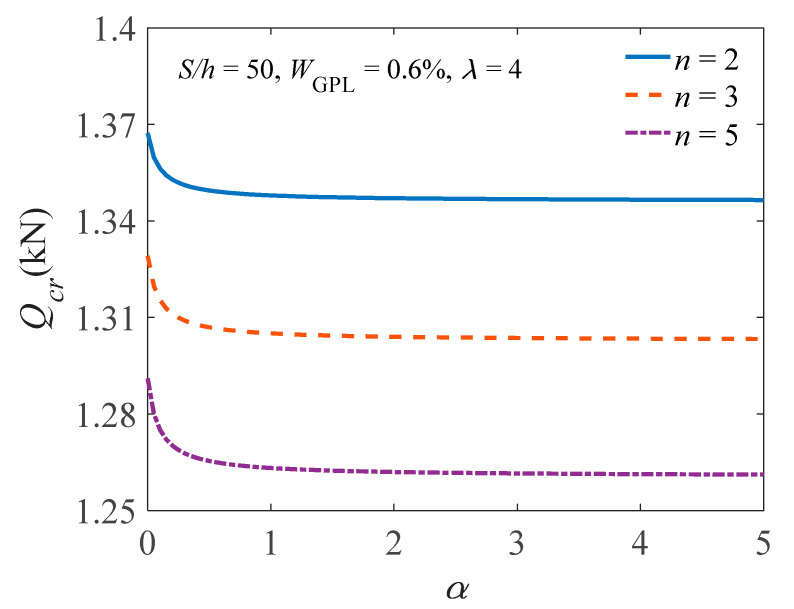
Effect of the flexibility of the rotational constraints on the buckling load.

Table 1 lists the buckling load of the arch having different GPL weight fractions. It is found that, by comparing with a pure epoxy arch (i.e., WGPL = 0.0%), GPLs have a remarkably reinforced effect on improving the buckling bearing capacity of the FG-GPLRC arch and the buckling load of the arch increases with an increase in WGPL. In addition, the effects of the geometry and size of GPLs on the buckling load are depicted in Figure 6. For *b*_GPL_/*t*_GPL_ = 10^3^, the reinforced effect of GPLs on improving the buckling bearing capacity of the FG-GPLRC arch becomes stable under different ratios of *a*_GPL_/*b*_GPL_. Figure 7 presents the flexibility of the rotational constraints on the buckling load of the FG-GPLRC arch. It is noted that, in all cases, the buckling load decreases as the flexibility of the rotational constraints increases, and the fixed FG-GPLRC arch has the highest one, as expected.

**Figure 8:**
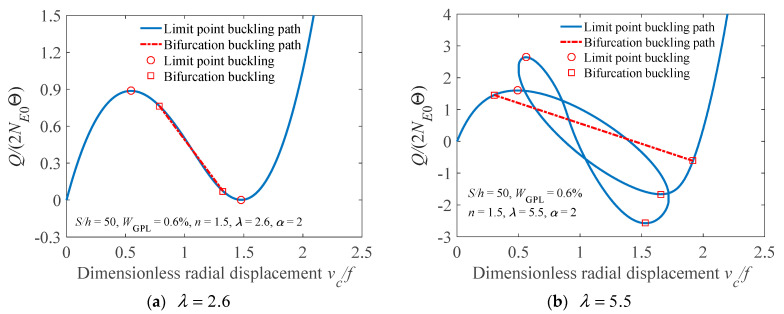
Nonlinear load-displacement curve of an elastically restrained FG-GPLRC arch.

Figure 8 displays the nonlinear equilibrium path of the FG-GPLRC arch in a load-displacement curve form to discuss the phenomenon of buckling mode switching. It is observed from Figure 8a that, when the arch has a smaller geometric parameter (e.g., λ=2.6), the bifurcation point is located behind the limit point, so the buckling mode of the arch is the limit point instability mode. However, when the FG-GPLRC arch has a higher geometric parameter (e.g., λ=5.5), its bifurcation point is located before the limit point, as shown in Figure 8b, leading to a bifurcation buckling mode of the arch instead of a limit point buckling mode. Accordingly, a critical geometric parameter $\lambda_{b2}$ is defined to identify the buckling mode of the arch, whose value is determined by solving Equations (27) and (34) at $\beta =\beta_{b}$ simultaneously. When the FG-GPLRC arch has $\lambda >\lambda_{b2}$, the buckling mode of the arch is bifurcation mode.

**Figure 9:**
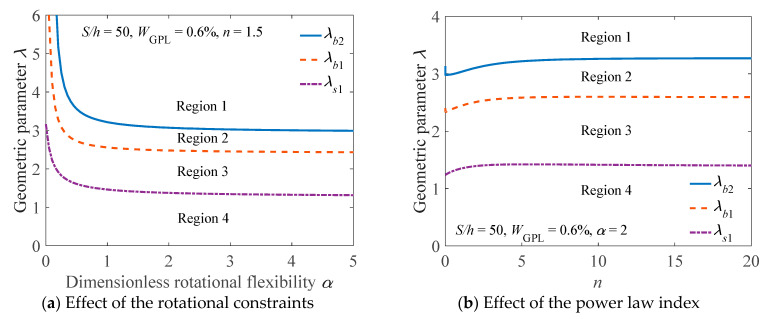
Critical geometric parameters for the buckling mode of an elastically restrained FG-GPLRC arch.

The effects of the rotational constraints and the power law index on the critical geometric parameters to distinguish the buckling mode are presented in Figure 9. As shown in this figure, in Region 1 ($\lambda >\lambda_{b2}$), the buckling mode of the arch is bifurcation mode. In Region 2 ($\lambda_{b1}\leq \lambda \leq \lambda_{b2}$), the buckling mode of the arch is either limit point instability mode or bifurcation mode, depending on which mode would occur first. In Region 3 ($\lambda_{s1}\leq \lambda <\lambda_{b1}$), the buckling mode of the arch is limit point instability mode. In Region 4 ($\lambda <\lambda_{s1}$), no buckling occurs in the arch. It should be mentioned that there is no solution to $\lambda_{b2}$ for α=0 which refer to the fixed support, it implies that the FG-GPLRC arch with fixed ends cannot buckle in a bifurcation mode.

**Figure 10:**
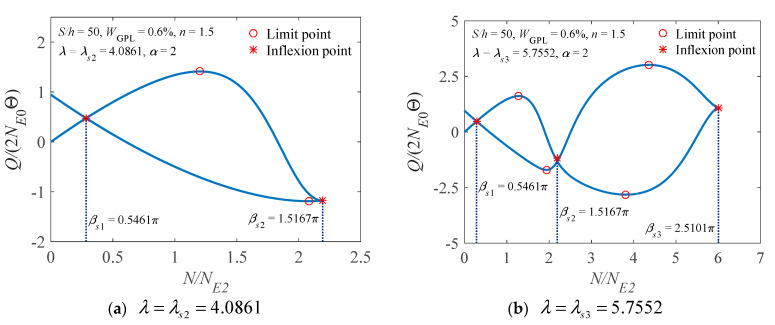
Nonlinear load-axial force curve of an elastically restrained FG-GPLRC arch.

**Figure 11:**
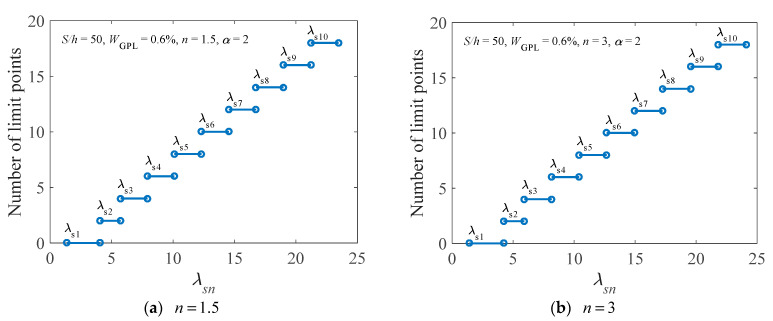
Number of limit points of an elastically restrained FG-GPLRC arch under different power law indices.

The nonlinear equilibrium paths of the FG-GPLRC arch, having the geometric parameters $\lambda =\lambda_{s2}=4.0861$ and $\lambda =\lambda_{s3}=5.7552$, are given in Figure 10. The number of limit points of the arch, in which the flexibility of the rotational constraints is set as two, is indicated in the figure. Note that we can determine the number of limit points within the bounds of successive inflexion points. When $\lambda <\lambda_{s1}$, there is no limit point in the nonlinear equilibrium path. When $\lambda_{s1}<\lambda <\lambda_{s2}$, there are two limit points in the nonlinear equilibrium path. When $\lambda_{s2}<\lambda <\lambda_{s3}$, there are four limit points in the nonlinear equilibrium path, and so on. Hence, the first 18 limit points and the corresponding critical geometric parameters $\lambda_{sn}$ of the arch with the power law index *n* = 1.5 and *n* = 3 are plotted in Figure 11 for further clarification.

## 6. Conclusions

In this paper, we studied the nonlinear buckling behavior of elastically supported FG-GPLRC arches with asymmetric distributed GPLs under a CPL. Analytical solutions for the limit point buckling, bifurcation buckling, and mode switching were derived. A finite element analysis was also employed to verify the present results, showing good accuracy of the present method for predicting the nonlinear buckling of the FG-GPLRC arch. According to the numerical studies, the influences of the distribution of GPLs, weight fractions, geometric configurations, as well as boundary restraints are discussed. It is found that the nonlinear buckling load of the FG-GPLRC arch decreases as the power law index or the flexibility of the rotational constraints increases. Switching of the buckling mode of the FG-GPLRC arch is quite sensitive to the power law index, the flexibility of rotational constraints, and the arch geometry. It is also found that, according to the present analytical method, the number of limit points in the nonlinear equilibrium path of the FG-GPLRC arch under a CPL can be easily determined.

## Figures and Tables

**Table 1:** Buckling loads of the FG-GPLRC arch having different weight fractions of distributed graphene platelets (GPLs).

*W*_GPL_ (%)	*n* = 0	*n* = 0.5	*n* = 1	*n* = 3
0.0	0.5088			
0.2	1.0157	0.8679	0.8313	0.7846
0.4	1.5227	1.2213	1.1454	1.0503
0.6	2.0300	1.5730	1.4567	1.3123

## Data Availability

Data can be obtained from the first author upon reasonable request.
